# Juvenile nasopharyngeal angiofibroma in a male of 16 years old. A case report

**DOI:** 10.1002/ccr3.3529

**Published:** 2020-12-05

**Authors:** Daniel Salame Waxman, Adelaido López Chavira, Martín Alberto Porras Jiménez, César López Hernández, Jorge Alberto Romo Magdaleno, Julio Eduardo López Montoy

**Affiliations:** ^1^ Department of Health Sciences School of Medicine Anahuac University Network Naucalpan de Juarez Mexico; ^2^ Undergraduate Internal Medical Teaching Department ABC Medical Center I.A.P. Centro Médico ABC Mexico City Mexico; ^3^ Department of Otolaryngology, Head and Neck Surgery Universidad del Ejercito y Fuerza Aerea Mexico City Mexico; ^4^ Department of Otolaryngology, Head and Neck Surgery Corporativo Hospital Satelite Naucalpan Mexico; ^5^ Department of Interventional Neuroradiology Hospital Angeles Lomas Huixquilucan Mexico; ^6^ Department of Otolaryngology, Head and Neck Surgery Hospital Central Militar Miguel Hidalgo Mexico

**Keywords:** benign head and neck tumor, endoscopic surgery, epistaxis, Juvenile nasopharyngeal angiofibroma

## Abstract

We present a multisystemic approach involving diverse specialists of a rare disease. Bringing into the perspective the importance of multidisciplinary work and complete patient knowledge in order to an adequate clinical practice and patient outcome.

## Graphical abstract

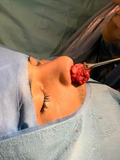

## 1 INTRODUCTION

Juvenile nasopharyngeal angiofibroma (JNA) is a tumor that occurs only in 0.05% of all head and neck tumors. We report a multisystemic approach combining study methods and collaboration with a radiologist for a 16‐year‐old male patient, diagnosed with a JNA measuring 2 × 3 × 5 cm with retarded diagnosis who received endoscopic surgery management.

Juvenile nasopharyngeal angiofibroma (JNA) was first described in 1897 by Chelius as a polyp that took place during the adolescence and early adulthood stage. This type of tumor occurs only in 0.05% of all head and neck tumors, it is benign, and as the name implies, it is installed in the nasopharynx, more specifically, in the sphenoid process of the palatine bone with the horizontal wing of vomer and on the roof of the pterygoid process of the sphenoid bone. Histologically, it is formed by a cylindrical and pavement epithelium with the characteristics of the epithelium found in the nasopharynx, below is a loose cellular tissue, and the neoformation itself is constituted by fibrous and highly vascularized tissue. JNA is almost exclusive to men, so it is believed that there is a certain hormonal relationship with the etiology that causes it, but still has not been proven. Despite the histological benignity, its clinical behavior can be malignant due to the growth along natural holes and fissures related to its site of origin, causing bone and vascular destruction, accompanied by large hemorrhages. The vessels lack a muscular layer, which leads to excessive bleeding with minimal manipulation or trauma of the tumor in question.^1^, ^2^, ^3^, ^4^, ^5^, ^6^

Its symptomatology is somewhat unspecific and generalized, characterized by a progressive nasal obstructive syndrome, usually unilateral, which ends up obstructing the choana. It is accompanied by night snoring, sleep apnea, headache, daytime oral breathing, and mild epistaxis. Eustachian tube dysfunction may occur if there is a nasopharyngeal component by the tumor. Symptoms usually become present 6 months to 1 year prior to diagnosis.^1^, ^2^, ^5^, ^6^

On physical examination with anterior rhinoscopy, it usually reveals a reddish lobed mass located in the back of the nasal cavity and the cavum; preoperative diagnosis is based on clinical and imaging characteristics; incisional biopsy can lead to massive bleeding and is not recommended routinely.^2^, ^3^

Within the diagnostic approach, computed tomography of the skull and sinuses is useful to show the extent of bone erosion due to the tumor, while magnetic resonance imaging is useful to delimit the margins of the tumor, especially if there is any intracranial extension.

Once the diagnosis is made, the JNA can be staged with Fisch and Radkowski classification.

Fisch classification:

- Stage I: Tumors limited to the nasal cavity and nasopharynx without bone destruction.
- Stage II: Tumors that invade the pterygomaxillary fossa and the sinuses with bone destruction.
- Stage III: Tumors that invade the infratemporal fossa, the orbit, and/or the parasellar region, but that maintain a lateral location with respect to the cavernous sinus.
- Stage IV: Tumors that invade the cavernous sinus, the area of the optic chiasma, and/or the pituitary fossa.

Radkowski Staging:

- Type IA—Limited to nose or nasopharynx.
- Type IB—Extension into at least one paranasal sinus.
- Type IIA—Minimal extension into sphenopalatine foramen. Includes minimal part of medial pterygomaxillary fossa.
- Type IIB—Full occupation of pterygomaxillary fossa with Holman‐Miller sign. Lateral or anterior displacement of maxillary artery branches. May have superior extension with orbital bone erosion.
- Type IIC—Extension through pterygomaxillary fossa into cheek, temporal fossa, or posterior to pterygoids.
- Type IIIA—Skull base erosion with minimal intracranial extension.
- Type IIIB—Skull base erosion with extensive intracranial extension ± cavernous sinus.

Regarding treatment before surgery, unilateral carotid angiography is necessary to evaluate the vascular supply of the angiofibroma and allow embolization of the main nutritional vessel, the internal maxillary artery. Occlusion of nutritional vessels reduces intraoperative bleeding, a major cause of morbidity, and may reduce tumor size. There are three main methods for embolization: transarterial, embolization with direct injection on the tumor, and a combination of the two. There is no preferred technique to perform an approach. ^1^, ^2^

It is customary to perform a more extensive approach such as lateral rhinotomy and degloving of the facial middle third. Open surgical methods are also used for locally advanced tumors, including those with intracranial involvement, optic nerve, or the internal carotid artery. The currently recommended treatment is surgical excision, which may or may not be accompanied by preoperative embolization.

Recently, thanks to the advances in endoscopic surgery, this method has been used as a therapeutic option in appropriate cases, since in larger lesions, extrusion of the tumor is preferred.

Radiation has been used in tumors that are not resectable or that are already advanced. It is a second option, although it is not needed in a large percentage of cases. ^2^, ^3^

Recurrence in this type of tumor does not respond to cell metastasis or dedifferentiation, but rather to the persistence of residual tissue in the surgical site or in sites that, due to their location, are not resectable during the first surgical period. The main sites where residual tissue is found are: pterygoid canal, pterygoid process, pterygopalatine foramen, nasopharynx, pterygopalatine fossa, sphenoid sinus, and infratemporal fossa (to name a few). ^4^

As mentioned earlier, the presentation of this type of tumor brings a clinical challenge to many medical specialties, making it an entity that should be brought into the picture when making a differential diagnosis with symptoms such as progressive nasal obstructive syndrome, night snoring, sleep apnea, headache, daytime oral breathing, and mild epistaxis.

Its clinical behavior makes the accurate diagnosis also as important, making the disease worse while time elapses from the symptoms’ onset to surgical treatment. Thanks to the modern devices to diagnose and treat this disease, it has been proven that the earlier this disease is diagnosed the prognosis is better in the long term.

With this case report, we aim to inform and prepare most clinicians into knowing this clinical entity and understanding its presentation, management, and follow‐up and to reduce the time lost from symptom onset to surgical management.

## 2 CASE HISTORY/EXAMINATION

A 16‐year‐old male patient with no important background history. His condition began 3 months ago, after a flu‐like illness, where he perceived a predominantly right nasal obstruction, with greater intensity at night, accompanied by hyposmia, occasional bloody rhinorrhea; as well as retronasal discharge, otic fullness, sometimes accompanied by mild epistaxis; later, right hemicranial headache, facial heaviness, sensation of retro‐ocular pressure is added to the movement; hyponasal voice and nighttime snoring. He reported having a cough and tiredness for a long time already. He also described having a feeling of instability to the movement, accompanied by nausea or sometimes vomiting for a month, and in the end a total nasal obstruction.

He went to a general practitioner doctor and a gastroenterologist who only gave him symptomatic management. Afterwards was referred to an otoneurologist who rules out any neurological alteration causing the symptoms. While performing rhinoscopy, obstruction in the right nostril is found. The patient reported occasional mild epistaxis in the left nostril, without reporting it to be uncontrollable, as well as having the flu 1 week prior, which was managed symptomatically. After 6 months, he was referred to us for medical consultation. After physical examination, we arrived at the following findings:

Using a 0mm 4mm endoscope support: Left septal deviation. Turbinate hypertrophy. A pinkish‐pearl right nasal tumor with a smooth, vascularized, ovoid surface that hangs from the back of the middle meatus and occupies the choana. Using a 70 degree endoscope retrogradely, nasopharyngeal occupation is seen incipiently. Right thick mucus is aspirated. Mouth, pharynx, and otoscopy without alterations. Hematic biometry and blood chemistry within normal values.

### 2.1 Differential diagnosis, investigations, and treatment

The differential diagnosis should be made with other nasopharyngeal tumors, such as choanal polyp, adenoid hyperplasia, nasopharyngeal tuberculosis, chordoma, craniopharyngioma, paraganglioma, teratoma, lymphomas, epidermoid carcinoma, adenoid cystic carcinoma, sarcomas, among others.

While talking specifically of the differences between the similar diagnosis and JNA is beyond the scope of this investigation, we can assure that the major differentiator of almost all the pathologies mentioned before is the chronic behavior they have, compared to the JNA, which is more of an acute and aggressive proliferative tumor, with invasion to the entire nasal cavity.

Simple skull tomography with contrast was made with the following findings:

Tumor in the depth of the nasal passage on the right side, apparently hypervascular that is insinuated in the sphenoparietal fossa, is related to a nasoangiofibroma as the first diagnostic possibility, other findings associated with inflammatory changes in the maxillary sinuses and hypertrophy in the mucosa of the lower cortices.

**Figure 1:**
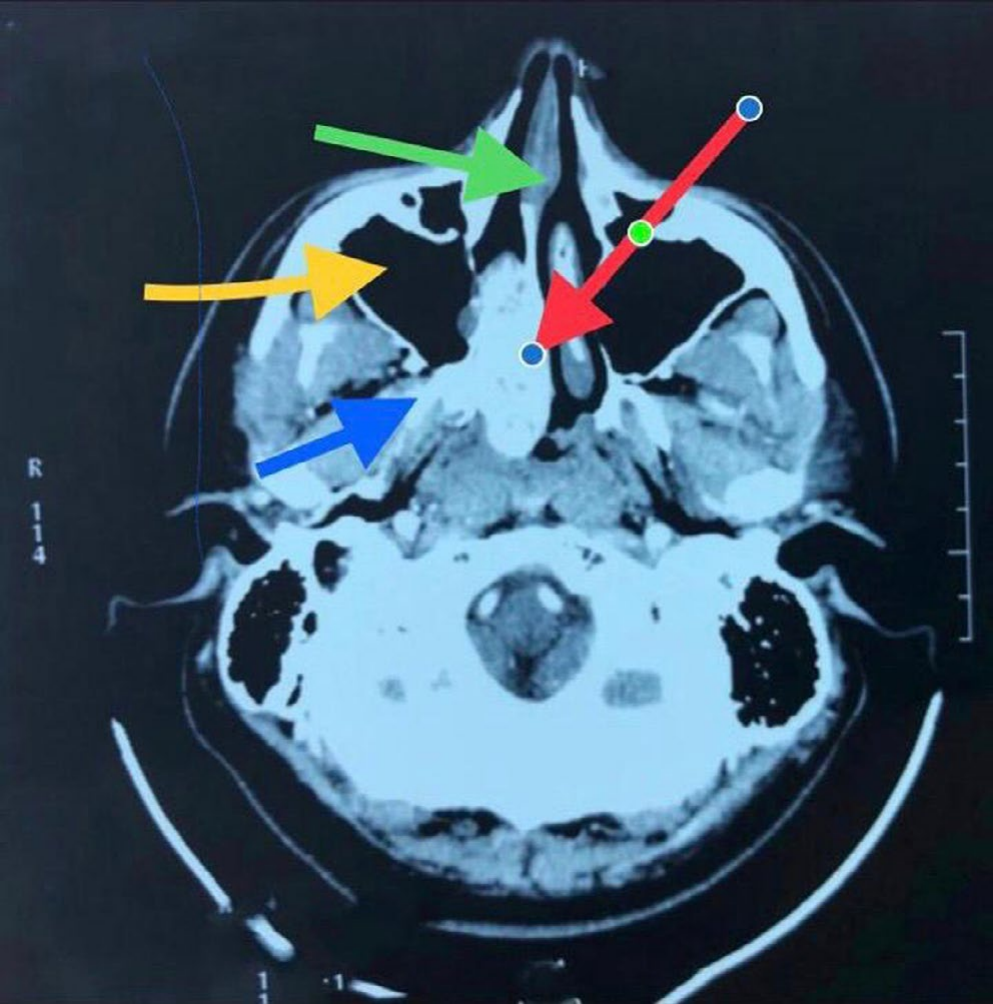
Axial cut of CT findings. Green Arrow: nasal septum. Red arrow: tumor in the nasal fossa and nasopharynx. Yellow arrow: right maxillary sinus. Blue arrow: tumor extension to the pterygopalatine fossa

Hypervascular lesion of the choana and right nasopharynx with slight pterygomaxillary extension. As a finding, there is a 2‐cm arachnoid cyst. (Figure 1).

**Figure 2:**
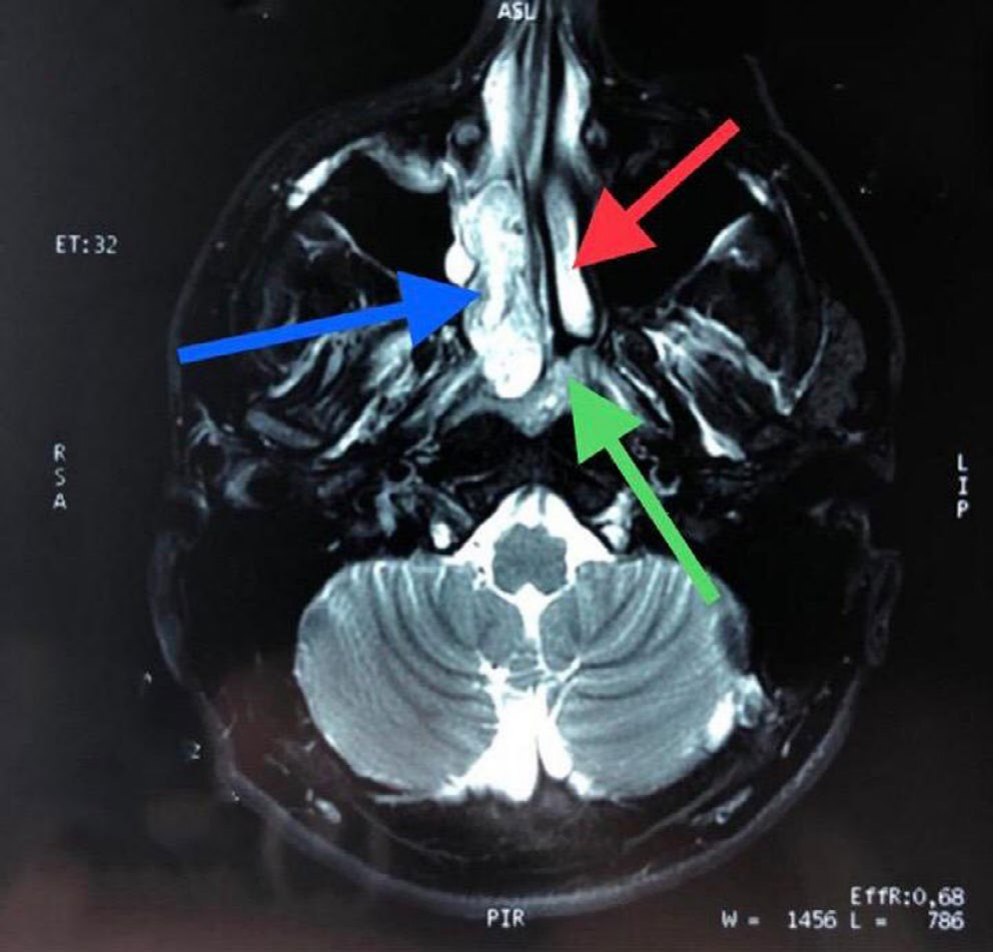
Axial cut of magnetic resonance findings. Red arrow: left middle turbinate. Blue arrow: tumor in the nostril and nasopharynx. Green arrow: sphenoid rostrum

Nuclear magnetic resonance imaging of the skull and facial mass: Tumor in the right nose and nasopharynx, without 2x3x5 cm extensions. Arachnoid cyst in the posterior fossa. Slight septal deviation. Concluding as probable diagnosis a right JNA (Stage I‐II/IV). (Figure 2).

**Figure 3:**
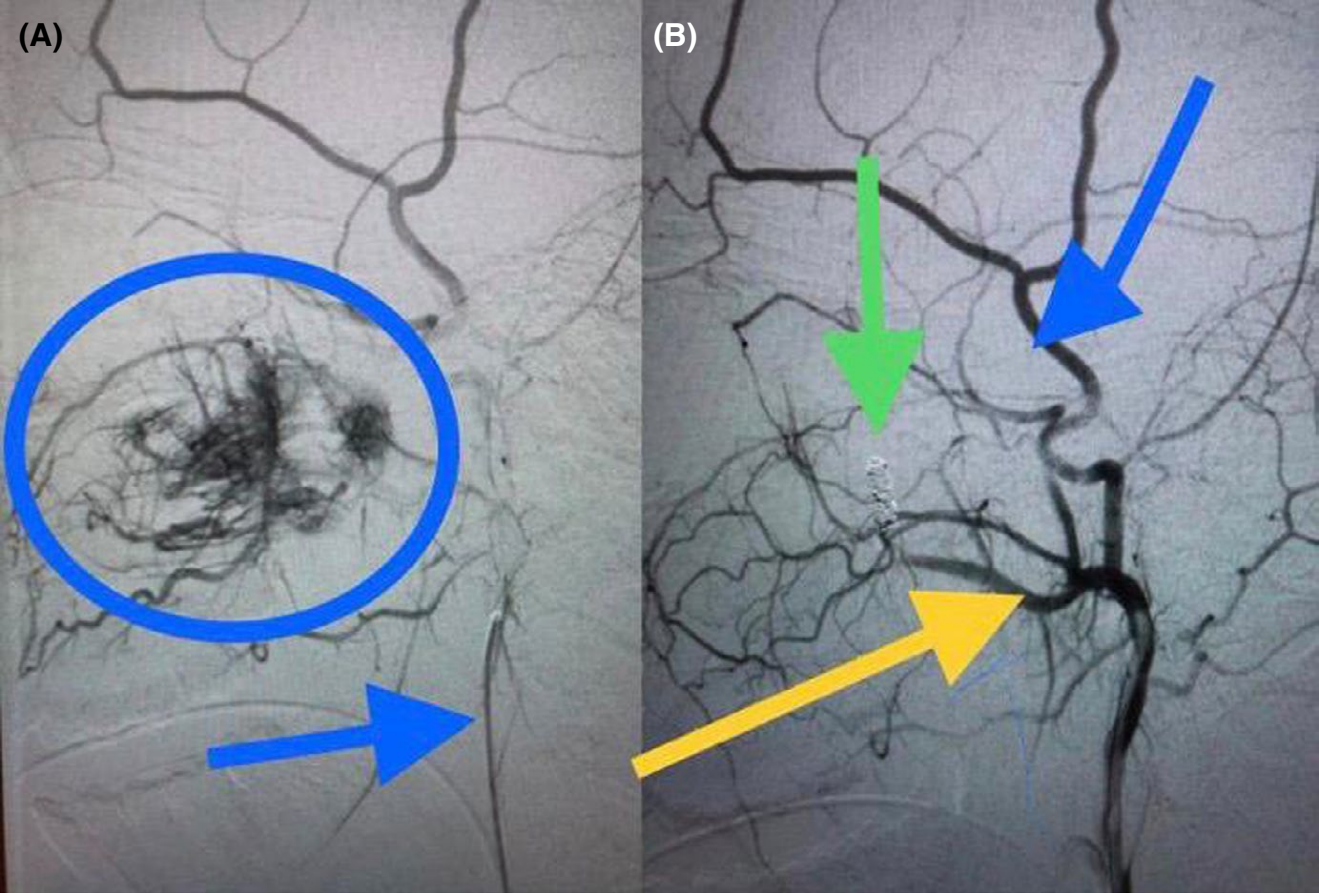
a and b‐ Carotid angiography a) Preembolization of supraselective approach of the right sphenopalatine artery. Blue circle: tumor prior to embolization. Blue arrow: Catheter in external carotid. b) Postembolization of supraselective approach of the right sphenopalatine artery. Green arrow: springs (coil) in tumor pedicle. Yellow arrow: sphenopalatine artery. Blue arrow: superficial temporal artery

On the day of hospital admission, a right selective angiography of external carotid arteries was made, with a supraselective approach performing an embolization of branches of the right sphenopalatine artery. During the surgical procedure, the following information was reported as findings: The known tumor shows irrigation dependent on the sphenopalatine artery through three main branches. The vessels show delayed staining, whose appearance is irregular and tortuous. After embolization, the result is occlusion of 100% of the blood supply. (Figure 3A and B).

**Figure 4:**
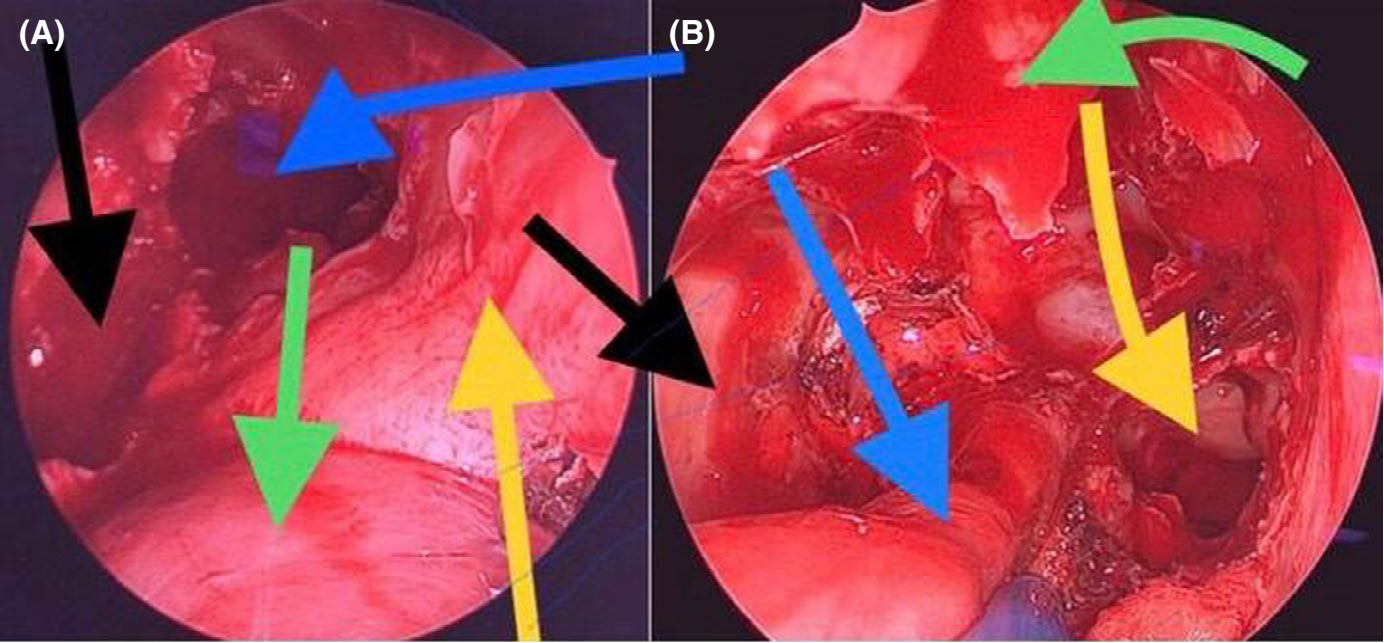
a and b ‐ Endoscopic resection a) Maxillary and sphenoid sinusotomy. Big blue arrow: sphenoid bone. Big black arrow: pterygopalatine fossa. Green straight arrow: nasal tumor. Yellow straight arrow: nasal septum. b) View and wide opening of maxillary, ethmoid, and sphenoid sinuses together with tumor pedicle. Green curved arrow: papyraceous lamina of the orbit. Small blue arrow: tumor pedicle. Yellow curved arrow: right choana. Black small arrow: posterior wall of the right maxillary sinus

**Figure 5:**
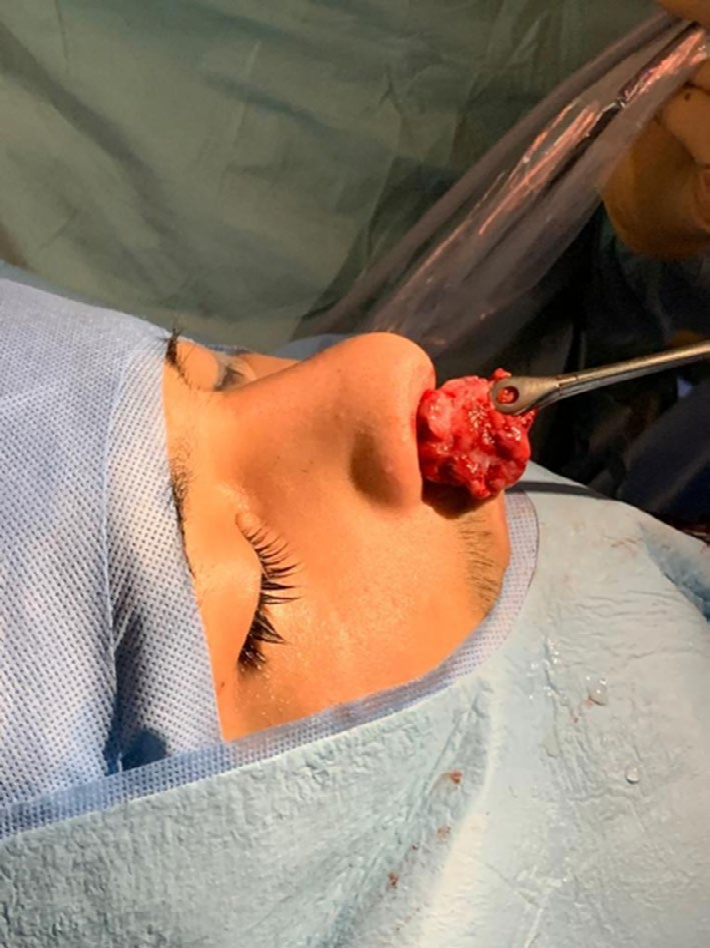
Extraction of the NJA

Two days later, the otorhinolaryngology surgical procedure was performed. With prior embolization, made 48 hours before with gelfoam and stainless steel coils by Seldinger technique, endoscopic surgery was performed as follows: Right anterior and posterior ethmoidectomy, opening of the recess and maxillary antrostomy III, sphenoidectomy (Figure 4A), cauterization of the lower corneal tail, cut of middle and upper turbinates; the medial part of the posterior wall of the right maxillary sinus was resected, identified, dissected, and coagulated (Figure 4B) and the sphenopalatine artery (already embolized) was cut, and a piece of the tumor was resected and extracted through the right nostril (Figure 5) (Video [Link], [Link]). Evista, Gelfoam, and Merocel were placed in the surgical bed. The patient evolved adequately and was discharged to his home two days after surgery.

### 2.2 Outcome and follow‐up

**Figure 6:**
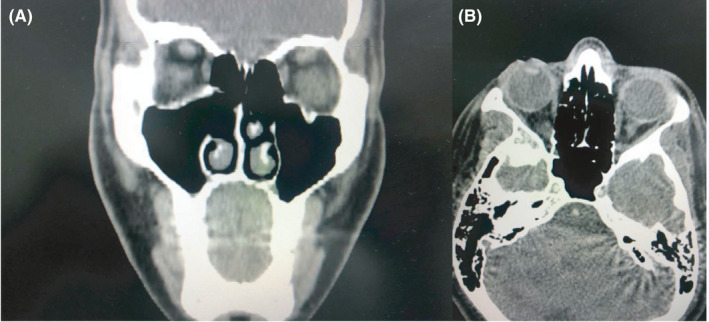
Axial and coronal cuts of CT scan, showing tumor‐free evolution after 9‐month control image

During the postoperative revision consultation in the office, a week after the procedure, tamponade removal and healing were performed, without complications. The patient is now 9 months tumor free of complications with an excellent outcome, and a CT made 9 months after surgical resection showed tumor‐free tissue (Figure 6).

## 3 DISCUSSION

JNA is a pathology with a low incidence; however, this should not be a factor for us to forget its possibility as a differential diagnosis in the presence of symptoms such as epistaxis, progressive nasal obstruction, sleep apnea, among others. In the case of our patient, a correct management was delayed for more than 6 months until an adequate diagnosis could be made after being evaluated by multiple specialists. ^1^, ^2^

Despite its benign nature, JNA is a rapidly growing and aggressive tumor, leading to bone destruction as well as invasion of adjacent structures. It has a well‐defined capsule that allows for correct manipulation and dissection during surgery. ^7^, ^8^, ^9^

The resection selected for our patient according to his Radkowski stage (I‐II/IV) was endoscopic surgical correction. This procedure is complicated but when performed correctly is an effective surgery, managing to limit recurrence with follow‐up to 24 months, attributing it to the advantageous multi‐angle view of the anatomical structures, enabling effective, atraumatic dissection using bipolar coagulation with gentle manipulation of the tumor. Among the decisive factors that were carried out correctly in our patient was a correct perforation of the bone invaded by the tumor, endonasal surgery combined with a preoperative embolization of the arterial supply of the tumor, thus achieving serious blood loss avoidance. ^7^, ^8^, ^9^, ^10^

In tumors that fall within the Radkowski IA‐IIB classification, endoscopic surgery remains an appropriate option. If the tumor extends laterally in the fossa infratemporalis or deep into the skull base, endoscopic surgery is no longer recommended. ^10^

## 4 CONCLUSION

As a rare disease, the JNA accounts for only 0.05% of all head and neck tumors. As mentioned before, the symptoms of the clinical behavior of this type of tumors can go from nonspecific to mild to moderate symptoms, making it a clinical challenge to most clinicians to have the JNA as a differential diagnosis. Regarding the treatment, thanks to the advances in technology, endoscopic resection is the preferred method in almost all the tumors, making the endoscopic resection of JNA a difficult but effective operation, with the preoperative embolization of the artery supply an important step on preventing big amounts of blood loss. With a well‐executed surgical management, and a correct clinical follow‐up, the prognosis for this type of tumors is generally favorable.

## CONFLICT OF INTEREST

None declared.

## AUTHOR CONTRIBUTIONS

Author 1: Head of the project, leader of the literature revision. Author 2: Head surgeon of the endoscopic procedure. Author 3: Head neuroradiologist that made the supraselective embolization. Author 4: Assistant neuroradiologist that made the supraselective embolization. Author 5: Assistant surgeon of the endoscopic procedure. Author 6: Assistant of the literature revision.

## ETHICS STATEMENT

Subscription of the protocol for evaluation by the ABC medical center teaching department and ethics committee. Follow‐up of the four principles of bioethics distinguished by Beauchamp and Childress: nonmaleficience, beneficence, autonomy, and justice. Follow‐up to the Declaration of Helsinki. Follow‐up of the agreements for research in humans established by the Good Medical Practice protocol. Follow‐up of the official norm in medicine number 004 regarding the clinical file. Follow‐up of the official norm in medicine number 012 on the criteria for the execution of research projects for health in human beings.

## INSTITUTIONAL REVIEW BOARD/ANIMAL CARE COMMITTEE APPROVAL

I certify that my institution or the appropriate regional institution has approved the protocol for any investigation involving humans or animals and that all investigations were conducted in conformity with the protocol and the ethical and humane principles of research. *“Clinical case reports”* reserves the right to contact your institution.

## PROTECTION OF HUMAN AND ANIMAL SUBJECTS

The authors declare that no experiments were performed on humans or animals for this study.

## CONFIDENTIALITY OF DATA

The authors have obtained written informed consent from the patients and/or subjects mentioned in the article. The corresponding author is in possession of this document.

## RIGHT TO PRIVACY AND INFORMED CONSENT

The authors declare that no patient data appear in this article.

## Supporting information

Supplementary Material

Supplementary Material

## Data Availability

The data that support the findings of this study are available on request from the corresponding author. The data are not publicly available due to privacy or ethical restrictions.
