# Supplementary material for: Juvenile nasopharyngeal angiofibroma in a male of 16 years old. A case report
Source: Clin Case Rep. 2020 Dec 5;9(1):355–61. doi: 10.1002/ccr3.3529 (PMC7813098; doi:10.1002/ccr3.3529)
Supplement: Supplementary file 2 — Supplementary Material [file CCR3-9-355-s002.docx]

Video 1 – Recording of the moment when the tumor pedicle is sectioned and cauterized.
